# Efficacy of a novel orally administered combination product containing sarolaner, moxidectin and pyrantel (Simparica Trio™) against induced infestations of five common tick species infesting dogs in the USA

**DOI:** 10.1186/s13071-020-3945-2

**Published:** 2020-03-01

**Authors:** Kristina Kryda, Sean P. Mahabir, Sara Chapin, Susan J. Holzmer, Laurel Bowersock, William R. Everett, John Riner, Lori Carter, David Young

**Affiliations:** 1 Zoetis, Veterinary Medicine Research and Development, 333 Portage Street, Kalamazoo, MI 49007 USA; 2 BerTek, Inc., P.O. Box 606, Greenbrier, AR 72058 USA; 3 Animal Health Innovations, Route 1, Box 435, Nowata, OK 74048 USA; 4 Stillmeadow, Inc., 12852 Park One Drive, Sugar Land, TX 77478 USA; 5 Young Veterinary Research Services, Inc., 7243 East Avenue, Turlock, CA 95389 USA

**Keywords:** Dog, Isoxazoline, Moxidectin, Oral combination, Pyrantel Pamoate, Sarolaner, Simparica Trio™, Tick

## Abstract

**Background:**

The efficacy of a novel oral combination product, Simparica Trio™, containing sarolaner, moxidectin and pyrantel was evaluated against five tick species that commonly infest dogs in the USA, *Amblyomma americanum*, *Amblyomma maculatum*, *Dermacentor variabilis*, *Ixodes scapularis* and *Rhipicephalus sanguineus*.

**Methods:**

Laboratory studies were conducted against two different strains of each tick species. In each study, 10 purpose-bred Beagle or mixed-breed dogs were randomly allocated to one of two treatment groups based on pre-treatment host-suitability tick counts. Dogs were infested with approximately 50 (45–55) unfed adult ticks on Days -2, 5, 12, 19, 26 and 33. On Day 0, dogs received either a single oral dose of Simparica Trio™ at the minimum label dose of 1.2 mg/kg sarolaner, 24 µg/kg moxidectin and 5 mg/kg pyrantel (as pamoate salt) or placebo. Tick counts were conducted at 48 h post-treatment and after each subsequent weekly re-infestation for *A. maculatum*, *D. variabilis*, *I. scapularis* and *R. sanguineus* studies and at 48 hours or at 72 h post-treatment and after weekly re-infestation in the first and second *A. americanum* studies, respectively.

**Results:**

No treatment-related adverse reactions occurred in any study. In all studies, placebo-treated dogs maintained infestations throughout the entire study duration, and dogs treated with Simparica Trio™ had significantly lower (*P* ≤ 0.0010) mean live tick counts than placebo-treated dogs at all time-points. Against *A. maculatum*, *D. variabilis*, *I. scapularis* and *R. sanguineus*, a single oral dose of Simparica Trio™ evaluated at 48 h post-treatment provided ≥ 98.9% efficacy against existing infestations, and within 48 h of re-infestation efficacy was ≥ 90.4% through at least Day 28 (except for *R*. *sanguineus* on Day 14 in a single study with an efficacy of 89.7%). Against *A. americanum*, Simparica Trio™ provided ≥ 99.4% efficacy at ≤ 72 h after treatment of existing infestations and maintained ≥ 98.4% efficacy at ≤ 72 h after re-infestation through at least Day 35.

**Conclusions:**

A single dose of Simparica Trio™ administered orally at the minimum label dosage of 1.2 mg/kg sarolaner, 24 µg/kg moxidectin and 5 mg/kg pyrantel provided treatment and control of the common tick species infesting dogs in the USA for at least one month.

## Background

Prevention and control of tick infestations remain major problems for dog owners within the USA and in many countries around the world [1–3]. Dogs infested with ticks can experience a broad range of clinical signs, ranging from local irritation and generalized hypersensitivity reactions to alopecia, tick paralysis and even anemia in extremely heavy infestations [3, 4]. In addition to being parasites themselves, ticks are known to be a primary source of pathogen transmission to animals, and are second only to mosquitoes, with respect to the transmission of vector-borne pathogens to humans [2, 5–8]. The five hard tick species used in the studies reported here, i.e. *Amblyomma americanum*, *Amblyomma maculatum*, *Dermacentor variabilis*, *Ixodes scapularis* and *Rhipicephalus sanguineus*, represent all genera (and ~ 50% of the species) known to commonly infest dogs and cats in the USA [3, 9, 10]. Additionally, all five are known to act as vectors for important canine pathogens, including *Anaplasma phagocytophilum* and *Borrelia burgdorferi* transmitted by *I. scapularis*; *Babesia canis*, *B. gibsoni* and *Ehrlichia canis* transmitted by *R. sanguineus*; *Ehrlichia ewingii* transmitted by *A. americanum*; *Hepatozoon americanum* transmitted by *A. maculatum*; and *Rickettsia rickettsii* transmitted by *D. variabilis* [3, 7, 9, 10]. The complexities of tick biology and ecology contribute to the difficulties associated with tick infestation prevention and control [4, 7]. The majority of ticks important in canine health, including the five species studied here, are hard ticks with multiple life stages (larva, nymph and adult) that each feed on a unique host. The high reproductive capacity of ticks adds to the problem, with suitable environmental conditions promoting the presence of large numbers of immature and adult ticks questing for a canine host [3]. After finding a suitable passing host, ticks will attach to the animal and begin feeding. The time period between attachment to the host and the transmission of pathogens, which may be as long as 24–48 hours, is a window of opportunity in the control of tick infestation and the prevention of tick-borne pathogen transmission, providing the chance to interrupt the tick life-cycle and the spread of disease [3, 11, 12].

Sarolaner belongs to a potent new class of ectoparasiticides (isoxazolines) that provides broad activity against fleas and ticks [13]. Previously efficacy against ticks has been demonstrated by sarolaner alone [14]. Recently, a novel oral combination product containing sarolaner in addition to moxidectin and pyrantel (Simparica Trio™, Zoetis, Parsipanny, NJ, USA) has been developed. Not only will Simparica Trio™ treat and control flea and tick infestations for one month in dogs [15], it will also treat roundworm and hookworm infections [16–18] and provide protection from lungworm [19] and heartworm disease [20].

The 10 studies reported here confirm the efficacy of Simparica Trio™ against five common tick species infesting dogs in the USA, *A. americanum*, *A. maculatum*, *D. variabilis*, *I. scapularis* and *R. sanguineus*.

## Methods

Ten laboratory studies were conducted to evaluate Simparica Trio™ against the following common tick species of the USA: *A. americanum* (lone star tick; Studies 1 and 2); *A. maculatum* (Gulf Coast tick; Studies 3 and 4); *D. variabilis* (American dog tick; Studies 5 and 6); *I. scapularis* (black-legged tick; Studies 7 and 8); and *R. sanguineus* (brown dog tick; Studies 9 and 10). Ticks were obtained from multiple laboratory-maintained colonies (one study used wild-caught ticks) so that two different USA strains of each tick species were tested. All studies were conducted in accordance with the CVM Guidance for Industry #85, Good Clinical Practice [21] and the World Association for the Advancement of Veterinary Parasitology (W.A.A.V.P.) guidelines for evaluating the efficacy of parasiticides for the treatment, prevention and control of flea and tick infestations on dogs and cats [22]. Masking was accomplished by separation of functions of study personnel. All personnel involved in making assessments of efficacy or safety were unaware of treatment assignments and dedicated personnel conducted the allocation of dogs and treatment dispensing, but did not conduct any other study observations.

### Animals

Ten dogs were included in each treatment group. Dogs were purpose-bred Beagle or mixed breed, uniquely identified, ranging from 7 months to 12 years of age and weighed between 5.3 and 35.1 kg at study initiation. Dogs were deemed to be in good health by a veterinarian and had demonstrated good tick retention prior to treatment. The animals were housed individually in indoor pens such that no physical contact was possible throughout the study and were fed a commercial dog food ration sufficient for a maintenance diet. Fresh water was available *ad libitum*. Study participants used separate protective clothing and equipment between each animal to prevent any cross-contamination between dogs.

### Design

Treatment day for all studies was designated as Day 0. The general health of each dog was assessed daily by a veterinarian for up to 3 weeks prior to treatment and for the duration of the study. Examinations included (but were not limited to) rectal temperature, thoracic auscultation, skin and hair assessment, and the overall physical health of the dog. Host suitability was determined prior to inclusion in the study and within 1 week of treatment on Day 0. All dogs were first examined to ensure they were free of ticks and then infested with 50 (± 5) viable, unfed ticks of the appropriate species. After 48 h (72 h for Study 2), live attached ticks were counted and removed, and the twenty dogs with the highest tick counts were selected for each study. Animals were allocated to treatments and pens according to a randomized complete block design, with room (if applicable) and block based on host suitability tick counts. Each dog was randomly allocated to a treatment group (Simparica Trio™ or placebo) in each block, and all dogs within a block were randomized to adjacent pens. Dogs were weighed, moved to their allocated pens and infested with ticks on Day -2. On Day 0, all dogs were assessed for overall health and then orally administered either the placebo or Simparica Trio™. Tick infestations (50 ± 5 viable ticks) were repeated on Days 5, 12, 19, 26 and 33, and live tick counts were performed 48 h (72 h for Study 2) after treatment on Day 0 and again after each infestation.

### Tick strains and infestations

Five tick species common in the USA were used in the ten studies, with two strains of USA origin used for each tick species. Outside of Study 8, which used wild-caught *I. scapularis* ticks collected within South Carolina during the two months prior to study start, ticks were obtained from laboratory-maintained colonies. These colonies were originally established using ticks collected from the field and had wild-caught ticks introduced into the tick colony within three years or less of the study start. For each infestation, 50 viable, unfed adult ticks with a sex distribution of approximately 1:1 were applied directly on each dog. Prior to infestation, three vials of ticks were randomly examined to demonstrate their correct preparation (number, sex ratio, viability, feed status). For Studies 2, 5, 8 and 10 the dogs were sedated prior to infestation; in all other studies dogs were not sedated.

### Treatment

On Day 0, dogs were treated orally with either placebo tablets containing inert formulation ingredients (vehicle) or the combination product (Simparica Trio™). Tablets were provided in varying strengths, such that a combination of tablets could be administered to ensure dogs were appropriately dosed to the minimum end of the label dose range. Each dog received one to three tablets of the combination product to provide as close as possible to the minimum label dosage of 1.2 mg/kg sarolaner (actual doses ranged from 1.2 to 1.6 mg/kg), 24 µg/kg moxidectin (actual doses ranged from 24.0 to 31.6 µg/kg) and 5 mg/kg pyrantel (as pamoate salt) (actual doses ranged from 5.0 to 6.6 mg/kg) or the equivalent number of placebo tablets. Body weights obtained on Day -2 were used for dose calculation. Feed was withheld for at least 12 h prior to treatment and animals were not fed again until at least 4 h post-treatment. All doses were administered by hand pilling to ensure accurate dosing. Dogs were observed for several minutes following dosing to ensure the entire treatment was swallowed and for up to 2 h post-dosing for any signs of emesis. Dogs were examined for general health and any reactions to treatment at 1, 3, 6 and 24 h after treatment.

### Tick counts

Tick counts were conducted by trained personnel either 48 h (9 studies) or 72 h (Study 2) after treatment on Day 0 or after each subsequent tick infestation. On each count day, the order in which the dogs were examined for tick counts was pre-defined by a randomization plan. Each dog was initially examined to identify readily visible ticks by pushing the dog’s fur against its natural nap. Then, an extra-fine tooth comb was used to comb the animal thoroughly and identify any previously missed ticks. Dogs were examined for a minimum of 10 min, and if any tick was encountered in the final minute, the combing was extended in 1-min increments until no additional ticks were encountered. All identified ticks were removed and examined to assess viability. Personnel conducting counts were masked to treatment assignments and changed gloves and protective clothing between dogs.

### Statistical analysis

The individual dog was the experimental unit and the primary endpoint was live tick count. Arithmetic means were used to summarize tick count by treatment group and time-point. The PROC MIXED procedure (SAS 9.4, Cary NC, USA) was used to analyse tick counts, using a mixed linear model with treatment group as a fixed effect and block, and error as random effects at each time-point. If multiple rooms were used in the study then the random effects included room, block within room, and error. Testing was two-sided at the significance level α = 0.05. Percent efficacy was calculated from arithmetic means using Abbott’s formula:

$${\text{\% Reduction}} = 100 \times \frac{{{\text{Mean count }}\left( {\text{placebo}} \right) {-} {\text{Mean count }}\left( {\text{treated}} \right)}}{{{\text{Mean count }}\left( {\text{placebo}} \right)}}$$

In Study 5 (*D. variabilis*), one treated dog was removed from the study after being diagnosed with a strangulating inguinal hernia on Day 9. Consequently, least squares means (rather than arithmetic means) and corresponding efficacies were used for data collected on Days 14–35 for this study [23].

## Results

### Efficacy

#### Amblyomma americanum

In studies using *A. americanum*, dogs in the placebo-treated groups maintained tick infestations for the entire study duration, with live ticks collected from all dogs at all counts (Table 1). Across the studies, mean live tick counts for dogs infested with *A. americanum* and given placebo were between 12.4–29.7 ticks and reflected between 24.8–59.4% of the number of ticks applied. In Study 1, Simparica Trio™ provided 100% efficacy against existing *A. americanum* infestations within 48 h post-treatment (Day 2). Following weekly re-infestations, efficacy in Study 1 remained at ≥ 99.5% for the entire 5 weeks after treatment. In Study 2, Simparica Trio™ provided 99.4% efficacy against *A. americanum* infestations within 72 h post-treatment (Day 3). Following weekly re-infestations, efficacy in Study 2 remained at ≥ 98.4% for the entire 5 weeks after treatment. Simparica Trio™-treated dogs had significantly lower mean live tick counts than placebo-treated dogs at all time-points (6.24 ≤ *t*_*df*_ ≤ 16.78, 9 ≤ *df* ≤ 18, *P* < 0.0001 for all tick counts) in both studies.

**Table 1:** Arithmetic mean tick counts and percent efficacy relative to placebo following one oral treatment with Simparica Trio™ for dogs with existing *Amblyomma americanum* infestations and after subsequent weekly re-infestations

Study	Tick strain	Day^a^	Placebo^b^		Simparica Trio™^b^		% Efficacy	Test statistic
			Mean	Range	Mean	Range		
1	Bertek Inc.^c^	2	19.0	3–32	0	0–0	100	*t*_(18)_ = 6.24, *P* < 0.0001
		7	23.1	14–38	0	0–0	100	*t*_(18)_ = 10.08, *P* < 0.0001
		14	20.7	13–33	0	0–0	100	*t*_(18)_ = 9.10, *P* < 0.0001
		21	23.1	14–37	0	0–0	100	*t*_(18)_ = 9.12, *P* < 0.0001
		28	20.9	13–29	0.1	0–1	99.5	*t*_(18)_ = 10.05, *P* < 0.0001
		35	29.7	20–44	0.1	0–1	99.7	*t*_(18)_ = 13.08, *P* < 0.0001
2	Ecto Services Inc^d^	3	17.7	12–27	0.1	0–1	99.4	*t*_(9.06)_ = 10.07, *P* < 0.0001
		8	23.7	12–35	0.1	0–1	99.6	*t*_(9.06)_ = 10.10, *P* < 0.0001
		15	16.1	5–25	0	0–0	100	*t*_(9)_ = 9.11, *P* < 0.0001
		22	13.9	7–23	0	0–0	100	*t*_(9)_ = 9.80, *P* < 0.0001
		29	12.4	9–16	0.2	0–1	98.4	*t*_(9)_ = 16.78, *P* < 0.0001
		36	14.4	4–25	0.1	0–1	99.3	*t*_(9.05)_ = 7.32, *P* < 0.0001

^a^First infestation on Day -2. Subsequent infestations on Day 5, 12, 19, 26 and 33. Tick counts were conducted at 48 (Study 1) or 72 (Study 2) hours post-treatment and subsequent re-infestation

^b^Treatment with placebo or Simparica Trio™ (minimum dose 1.2 mg/kg sarolaner, 24 µg/kg moxidectin and 5 mg/kg pyrantel (as pamoate salt)) occurred on Day 0

^c^Colony originated from ticks field-collected in Greenbrier, AR, USA, in 2009 and was last supplemented with wild-caught ticks ~ 3 years prior to study start

^d^Colony originated from ticks field-collected in Stillwater, OK, USA, in 2011 and was last supplemented with field-collected ticks ~ 4 months prior to study start

#### Amblyomma maculatum

Dogs infested with *A. maculatum* and treated with the placebo maintained tick infestations throughout the duration of both studies, with live ticks collected from all control dogs at all counts except for one control dog in Study 4 which had no live ticks collected on Day 7 (Table 2). This dog did have live tick counts of 14–32 for all other days. Mean live tick counts for placebo-treated dogs in both studies were between 23.1–35.1, representing 46.2–70.2% of ticks used for infestation. When used to treat existing infestations, Simparica Trio™ provided 100% efficacy in Study 3 and 99.1% efficacy in Study 4 within 48 h post-treatment (Table 2; Day 2 counts). Following weekly re-infestations, efficacy in both studies was ≥ 90.4% for 28 days. Dogs treated with Simparica Trio™ had significantly lower arithmetic mean live tick counts than placebo-treated dogs at all time-points in both studies (Study 3, 4.78 ≤ *t*_*df*_ ≤ 10.61, 9 ≤ *df* ≤ 9.06, *P* ≤0.0010 for all tick counts; Study 4, 5.23 ≤ *t*_*df*_ ≤ 9.14, 9 ≤ *df* ≤ 17, *P* ≤0.0005 for all tick counts).

**Table 2:** Arithmetic mean tick counts and percent efficacy relative to placebo following one oral treatment with Simparica Trio™ for dogs with existing *Amblyomma maculatum* infestations and after subsequent weekly re-infestations

Study	Tick strain	Day^a^	Placebo^b^		Simparica Trio™^b^		% Efficacy	Test statistic
			Mean	Range	Mean	Range		
3	Oklahoma State University^c^	2	30.0	3–74	0	0–0	100	*t*_(9)_ = 4.78, *P* = 0.0010
		7	33.8	14–50	0.1	0–1	99.7	*t*_(9)_ = 8.12, *P* < 0.0001
		14	30.1	15–44	0.1	0–1	99.7	*t*_(9.02)_ = 10.61, *P* < 0.0001
		21	32.6	12–47	0.2	0–2	99.4	*t*_(9.05)_ = 8.58, *P* < 0.0001
		28	23.6	6–42	0.3	0–2	98.7	*t*_(9.06)_ = 6.27, *P* = 0.0001
		35	23.1	6–45	0.7	0–2	97.0	*t*_(9)_ = 6.31, *P* = 0.0001
4	Texas A&M University^d^	2	35.1	11–50	0.3	0–2	99.1	*t*_(9.05)_ = 8.51, *P* < 0.0001
		7	26.3	0–45	0.3	0–2	98.9	*t*_(9.04)_ = 5.43, *P* = 0.0004
		14	33.9	14–54	0	0–0	100	*t*_(17)_ = 8.14, *P* < 0.0001
		21	28.9	13–46	0.7	0–4	97.6	*t*_(9)_ = 7.24, *P* < 0.0001
		28	25.0	13–37	2.4	0–15	90.4	*t*_(9)_ = 9.14, *P* < 0.0001
		35	25.7	6–51	6.1	0–18	76.3	*t*_(9)_ = 5.23, *P* = 0.0005

^a^First infestation on Day -2. Subsequent infestations on Day 5, 12, 19, 26 and 33. Tick counts were conducted at 48 hours post-treatment and weekly re-infestation

^b^Treatment with placebo or Simparica Trio™ (minimum dose 1.2 mg/kg sarolaner, 24 µg/kg moxidectin and 5 mg/kg pyrantel (as pamoate salt)) occurred on Day 0

^c^Colony originated from ticks field-collected in Stillwater, OK, USA, in 1991 and was last supplemented with wild-caught ticks ~ 15 months prior to study start

^d^Colony originated from ticks field-collected in Refugio Co., TX, USA, in the mid-1980s and was last supplemented with wild-caught ticks ~ 2 years prior to study start

#### Dermacentor variabilis

Placebo-treated dogs infested with *D. variabilis* maintained tick infestations for the entire duration of both Studies 5 and 6. Live ticks were collected from all control dogs at each tick count, with mean tick counts ranging from 19.7 to 39.8 (39.4–79.6% of ticks infested) (Table 3). Efficacy of Simparica Trio™ against existing *D. variabilis* infestations was high, being 99.7% in Study 5 and 98.9% in Study 6, 48 h after treatment. Efficacy against subsequent weekly re-infestations remained above 92% for 28 days and was 86.7% on Day 35 in Study 5. In Study 6, efficacy remained at ≥ 98.3% for the entire 35 days after treatment. In both studies, live tick counts were significantly lower for Simparica Trio™-treated dogs compared to placebo-treated dogs on all days (7.79 ≤ *t*_*df*_ ≤ 23.79, 8.06 ≤ *df* ≤ 9.17, *P* <0.0001 for all tick counts).

**Table 3:** Arithmetic mean tick counts and percent efficacy relative to placebo for dogs following one oral treatment with Simparica Trio™ with existing *Dermacentor variabilis* infestations and after subsequent weekly re-infestations

Study	Tick strain	Day^a^	Placebo^b^		Simparica Trio™^b^		% Efficacy	Test statistic
			Mean	Range	Mean	Range		
5	Oklahoma State University^c^	2	34.0	20–44	0.1	0–1	99.7	*t*_(9.02)_ = 11.57, *P* < 0.0001
		7	39.8	29–50	0.4	0–3	99.0	*t*_(9)_ = 20.32, *P* < 0.0001
		14^e^	39.1	30–46	0.1	0–1	99.7	*t*_(8.06)_ = 23.79, *P* < 0.0001
		21^e^	30.4	16–42	1.1	0–6	96.4	*t*_(8.06)_ = 11.16, *P* < 0.0001
		28^e^	31.2	23–42	2.3	0–19	92.6	*t*_(8.59)_ = 15.04, *P* < 0.0001
		35^e^	31.6	24–41	4.2	0–27	86.7	*t*_(8.68)_ = 10.59, *P* < 0.0001
6	Ecto Services Inc^d^	2	26.8	16–42	0.3	0–2	98.9	*t*_(9.17)_ = 11.91, *P* < 0.0001
		7	19.7	14–31	0.2	0–1	99.0	*t*_(9.09)_ = 10.55, *P* < 0.0001
		14	28.7	10–48	0.5	0–2	98.3	*t*_(9)_ = 8.16, *P* < 0.0001
		21	30.4	20–38	0	0–0	100	*t*_(9)_ = 17.67, *P* < 0.0001
		28	31.1	22–41	0	0–0	100	*t*_(9)_ = 14.56, *P* < 0.0001
		35	28.0	12–51	0.4	0–2	98.6	*t*_(9)_ = 7.79, *P* < 0.0001

^a^First infestation on Day -2. Subsequent infestations on Day 5, 12, 19, 26 and 33. Tick counts were conducted at 48 hours post-treatment and weekly re-infestation

^b^Treatment with placebo or Simparica Trio™ (minimum dose 1.2 mg/kg sarolaner, 24 µg/kg moxidectin and 5 mg/kg pyrantel (as pamoate salt)) occurred on Day 0

^c^Colony originated from ticks field-collected in Stillwater, OK, USA, in 1972 and was last supplemented with wild-caught ticks the same year as the study started

^d^Colony started from ticks field-collected in Stillwater, OK, USA, in 2015 (one year prior to study start)

^e^Study 5: Time-points with missing observations (Days 14–35) used least squares means and corresponding % efficacies

#### Ixodes scapularis

Control dogs infested with *I. scapularis* maintained tick infestations throughout the course of both Studies 7 and 8, with live ticks removed from each dog at each tick count. Across both studies, mean live tick counts for control dogs were between 13.8–27.2, representing 27.6–54.4% of the 50 ticks used for infestation of each dog (Table 4). Simparica Trio™ provided 100% efficacy against existing infestations in both studies at 48 h post-treatment. Following weekly re-infestations, efficacy was 100% for all tick count days except Day 21, when it remained ≥ 95.1% across both studies. The < 100% efficacy recorded in both studies on Day 21 were due to (i) ten live ticks collected from one Simparica Trio™-treated dog in Study 7 (95.1% efficacy) and (ii) a single tick removed from one dog in Study 8 (99.6%). The mean number of live ticks collected from Simparica Trio™-treated dogs was significantly lower than for control dogs at each tick count in both studies (6.64 ≤ *t*_*df*_ ≤ 21.84, 9 ≤ *df* ≤ 18, *P* < 0.0001 for all tick counts).

**Table 4:** Arithmetic mean tick counts and percent efficacy relative to placebo following one oral treatment with Simparica Trio^™^ for dogs with existing *Ixodes scapularis* infestations and after subsequent weekly re-infestations

Study	Tick strain	Day^a^	Placebo^b^		Simparica Trio^™b^		% Efficacy	Test statistic
			Mean	Range	Mean	Range		
7	Oklahoma State University^c^	2	21.9	14–34	0	0–0	100	*t*_(17)_ = 12.10, *P* < 0.0001
		7	13.8	3–20	0	0–0	100	*t*_(17)_ = 9.34, *P* < 0.0001
		14	19.6	5–31	0	0–0	100	*t*_(18)_ = 8.72, *P* < 0.0001
		21	20.6	13–34	1.0	0–10	95.1	*t*_(9)_ = 8.63, *P* < 0.0001
		28	21.6	9–37	0	0–0	100	*t*_(17)_ = 6.64, *P* < 0.0001
		35	23.2	10–35	0	0–0	100	*t*_(18)_ = 9.85, *P* < 0.0001
8	Wild-caught ticks^d^	2	26.7	20–36	0	0–0	100	*t*_(9)_ = 16.02, *P* < 0.0001
		7	27.2	22–35	0	0–0	100	*t*_(9)_ = 19.47, *P* < 0.0001
		14	25.5	19–32	0	0–0	100	*t*_(9)_ = 20.27, *P* < 0.0001
		21	26.6	22–34	0.1	0–1	99.6	*t*_(9)_ = 21.84, *P* < 0.0001
		28	24.8	19–33	0	0–0	100	*t*_(9)_ = 17.18, *P* < 0.0001
		35	26.8	19–37	0	0–0	100	*t*_(9)_ = 17.73, *P* < 0.0001

^a^First infestation on Day -2. Subsequent infestations on Day 5, 12, 19, 26 and 33. Tick counts were conducted at 48 hours post-treatment and weekly re-infestation

^b^Treatment with placebo or Simparica Trio^™^ (minimum dose 1.2 mg/kg sarolaner, 24 µg/kg moxidectin and 5 mg/kg pyrantel (as pamoate salt)) occurred on Day 0

^c^Colony originated from ticks field-collected in Stillwater, OK, USA, in 1991 and was last supplemented with wild-caught ticks 10 months prior to study start

^d^Wild-caught adult ticks collected in South Carolina, USA, during the two months prior to study start

#### Rhipicephalus sanguineus

In both Studies 9 and 10, live ticks were removed from control dogs infested with *R. sanguineus* with mean tick counts ranging from 11.7 to 34.7, representing 23.4–69.4% of the 50 ticks used for infestation (Table 5). Efficacy of Simparica Trio™ against existing *R. sanguineus* infestations was ≥ 99.6% at 48 h post-treatment in both studies, with one dog in each study having a single live tick on Day 2. Following weekly re-infestations in Study 10, efficacy of Simparica Trio™ was ≥ 97.0% for 28 days and remained at 94.0% on Day 35. Against weekly re-infestations in Study 9, Simparica Trio™ displayed ≥ 94.2% efficacy on all days except Day 14; on that day, 12 live ticks were collected from a single dog, resulting in an efficacy of 89.7%. No ticks were collected from this dog on any other day. For both studies, the mean number of live ticks collected from Simparica Trio™-treated dogs was always significantly lower than for control dogs (5.50 ≤ *t*_*df*_ ≤ 22.01, 9 ≤ *df* ≤ 18, *P* < 0.0001 for all tick counts).

**Table 5:** Arithmetic mean tick counts and percent efficacy relative to placebo for dogs following one oral treatment with Simparica Trio^™^ with existing *Rhipicephalus sanguineus* infestations and after subsequent weekly re-infestations

Study	Tick strain	Day^a^	Placebo^b^		Simparica Trio™^b^		% Efficacy	Test statistic
			Mean	Range	Mean	Range		
9	Bertek Inc^c^	2	23.8	12–40	0.1	0–1	99.6	*t*_(9.02)_ = 7.59, *P* < 0.0001
		7	17.3	9–28	1.0	0–10	94.2	*t*_(9)_ = 8.24, *P* < 0.0001
		14	11.7	3–17	1.2	0–12	89.7	*t*_(18)_ = 5.98, *P* < 0.0001
		21	15.1	4–26	0	0–0	100	*t*_(18)_ = 7.00, *P* < 0.0001
		28	14.6	4–26	0	0–0	100	*t*_(17)_ = 6.46, *P* < 0.0001
		35	12.8	4–26	0	0–0	100	*t*_(17)_ = 5.50, *P* < 0.0001
10	Ecto Services Inc^d^	2	34.7	29–46	0.1	0–1	99.7	*t*_(9)_ = 22.01, *P* < 0.0001
		7	29.4	19–43	0.1	0–1	99.7	*t*_(9.03)_ = 12.80, *P* < 0.0001
		14	21.1	15–30	0.1	0–1	99.5	*t*_(9)_ = 13.95, *P* < 0.0001
		21	23.1	13–39	0.7	0–3	97.0	*t*_(9.46)_ = 8.89, *P* < 0.0001
		28	24.6	20–37	0.2	0–1	99.2	*t*_(9)_ = 16.78, *P* < 0.0001
		35	24.9	19–33	1.5	0–6	94.0	*t*_(12.3)_ = 13.68, *P* < 0.0001

^a^First infestation on Day -2. Subsequent infestations on Day 5, 12, 19, 26 and 33. Tick counts were conducted at 48 hours post-treatment and weekly re-infestation

^b^Treatment with placebo or Simparica Trio^™^ (minimum 1.2 mg/kg sarolaner, 24 µg/kg moxidectin and 5 mg/kg pyrantel (as pamoate salt)) occurred on Day 0

^c^Colony originated from ticks field-collected in Greenbrier, AR, USA, in 2006 and was last supplemented with wild-caught ticks ~ 2 years prior to study start

^d^Colony originated from ticks collected in 1991 from dogs in Stillwater, OK, USA, and was obtained from Professional Laboratory and Research Service, Inc (PLRS), Corapeake, NC, USA. Last supplementation of the colony with wild-caught ticks was 2–3 months prior to study start

### Health observations

No post-treatment abnormal health observations were recorded from any dogs in Studies 1, 4, 7, 9 or 10. Abnormal health observations were recorded for a total of nine dogs from the remaining five studies, and none were deemed to be associated with treatment. Recorded events included mild reactions to tick infestation, including localized dermal response, target lesion due to tick bite and mild swelling (five dogs; four placebo-treated and one combination product-treated), otitis externa and dermatitis (one placebo-treated), cherry eye (one combination product-treated) and mammary mass (one combination product-treated). As noted in the Methods section, a single placebo-treated dog was removed from Study 5 after being diagnosed with a strangulating inguinal hernia on Day 9.

## Discussion

The ten studies reported here confirm the high and consistent efficacy of Simparica Trio™ in treating existing infestations and controlling re-infestations of five common and important dog tick species in the USA. Treating dogs with a single oral dose of Simparica Trio™ 2 days after infestation resulted in 100% removal of existing *I. scapularis* infestations and ≥ 98.9% removal of *A. maculatum*, *D. variabilis* and *R. sanguineus* infestations within 48 hours of treatment. Against *A. americanum*, Simparica Trio™ provided ≥ 99.4% effectiveness at ≤ 72 hours after treatment of existing infestations. In all ten studies, Simparica Trio™ continued to significantly (5.50 ≤ *t*_*df*_ ≤ 22.01, 9 ≤ *df* ≤ 18, *P* ≤0.0010) reduce live tick numbers of all species following re-infestation for at least 28 days. Efficacy of Simparica Trio™ against re-infestations of *A. maculatum*, *D. variabilis*, *I. scapularis* and *R. sanguineus* at Day 28 at 48 hours was ≥ 90.4%, with 96.3% of dogs (77 out of 80) having 2 or fewer ticks and 86.3% of dogs being completely protected. Against re-infestations of *A. americanum*, Simparica Trio™ provided ≥ 98.4% efficacy at 72 hours for at least 5 weeks. These data show Simparica Trio™ provides dogs with highly effective treatment of existing tick infestations and reliable protection from re-infestation between monthly treatments.

Recently, ecological changes due to warmer temperatures and human modification in conjunction with the increased movement of hosts (human and animal) both within and across country borders have contributed to elevated tick densities in certain areas and the appearance of some species in new habitats [1, 10, 24, 25]. Not surprisingly, the epidemiology of tick-borne diseases in animals also appears to be shifting, with some pathogens appearing to be re-emerging and others being reported in new geographic locales and host populations [1, 2, 5, 7, 10, 26]. The rapid killing of ticks and the prevention of attachment and feeding are important in reducing infestation-associated clinical signs and preventing tick-borne disease transmission. Following host attachment, 24–48 hours is usually needed before ticks are able to transmit tick-borne pathogens to hosts [11, 27]. After attaching, ticks first enter a slow feeding phase that lasts for 3–5 days before moving into a more rapid state of feeding [1]. The first few days after host attachment is a critical time in tick biology and provides a window of opportunity for intervention. Simparica Trio™ administered to dogs has shown rapid efficacy against *I. scapularis*, beginning to kill ticks within 8 hours of administration against an existing infestation and providing ≥ 94.2% effectiveness against re-infestations within 24 hours for 28 days [28]. While the studies reported here did not focus on speed of kill, it is important to note that the efficacy of Simparica Trio™ was ≥ 98.9% at the first post-treatment evaluations for all five tick species. Furthermore, of the 20 post-treatment evaluations conducted for each species over the course of 1 month (Day 28/29), at least 85% showed efficacy ≥ 99%, suggesting monthly administration of Simparica Trio™ will effectively protect dogs from recurring tick infestations and may reduce the transmission of tick-borne diseases.

While numerous parasite preventives are currently available for use in dogs, extensive protection against both ecto- and endoparasites usually requires multiple medications often with varying administration routes and directions [29]. Recent research in both the USA and Europe shows owner compliance in the prevention and treatment of internal and external parasites frequently falls short of expert recommendations [30–34]. Within the USA, 73% of dog owners in one survey believed their dog should receive year-round flea and tick preventives but only 13% actually purchased sufficient medication to achieve year-round protection [32], and owner compliance in the correct administration of canine heartworm preventive is documented as well below 100% [30, 35]. The use of multiple medications may add further hurdles for owners; an analysis of clinic transaction records for 231,565 dogs receiving flea and tick medication showed approximately 66% of owners purchased insufficient protection to provide the veterinarian’s recommended year-round protection [31]. If increased owner compliance, and consequently improved pet health, is to be achieved, then newly developed products need to provide comprehensive protection and thereby reduce the treatment burden on owners. Combining the proven preventive moxidectin [36–39] and anthelmintic pyrantel [40–42] with sarolaner, Simparica Trio™ provides a means of aligning ectoparasite treatment and control with currently available endo-parasiticides, addressing the needs of pet owners by providing broad-spectrum parasite protection in a single convenient product.

## Conclusions

A single oral dose of the combination product (Simparica Trio™) administered at the minimum label dose of 1.2 mg/kg sarolaner, 24 µg/kg moxidectin and 5 mg/kg pyrantel (as pamoate salt), displayed robust efficacy (≥ 98.9%) against existing infestations of five common USA tick species infesting dogs within 48 to 72 hours of administration. Efficacy at 48 hours against re-infestation by *A. maculatum*, *D. variabilis*, *I. scapularis* and *R. sanguineus* was ≥ 90.4% at Day 28. Efficacy at ≤ 72 hours against re-infestation by *A. americanum,* was ≥ 98.4% for at least 35 days. At all observations, Simparica Trio™ significantly reduced live tick numbers of all species compared to controls. Simparica Trio™ administered monthly provides owners and veterinarians with a highly effective means of treating and controlling tick infestations on dogs.

## Data Availability

All relevant data supporting the conclusions of this article are included within the article.
